# Differences in the course of Recurrent Depressive Disorder and Bipolar Affective Disorder occurring with apathetic depression

**DOI:** 10.1192/j.eurpsy.2026.11188

**Published:** 2026-07-31

**Authors:** S. Sorokin, A. Churkina, M. Popov

**Affiliations:** Department for the Study of Endogenous Mental Disorders and Affective States, Mental health research center, Moscow, Russian Federation

## Abstract

**Introduction:**

Apathy is one of the most common phenomena in the clinical structure of endogenous affective disorders. Apathy significantly impacts patients’ social and occupational functioning and is often an unfavorable prognostic indicator.

**Objectives:**

To identify differences in the dynamics of recurrent depressive disorder and bipolar affective disorder occurring with apathetic depression.

**Methods:**

The study included patients with apathetic depression within unipolar (19 cases) and bipolar (24 cases) dynamics of affective disorder (ICD-10 codes F31, F33), aged 27 to 60 years (average 34.2 years), with a disease duration of at least 9 years. Depression severity was moderate or severe (the Hamilton Rating Scale for Depression score >14). A total of 97 depressive episodes in 43 patients were analyzed.

**Results:**

Dynamics of recurrent depressive and bipolar affective disorder showed differences concerning frequency and development mechanism of affective episodes, duration and intensity of manifest and recurrent affective states, intensity of depressions, quality of remissions and other features. Recurrent depressive disorder was characterized by later onset (often after age 28), longer depressive episodes (p < 0.05), lower severity of depressive symptoms, and a simpler, more typical symptom structure. Depressive episodes often developed reactively (86.2% of all studied episodes). Patients in this group predominantly had hyperthymic or harmonious personality features. Poor prognosis was associated with prolonged depressive episodes (often exceeding six months). Bipolar disorder showed earlier onset (before age 25) (p < 0.01), lower severity of initial affective episodes with progressive worsening over time, more frequent episode recurrence, shorter remissions, and an autochthonous (spontaneous) mechanism of depression development (p < 0.01). Atypical depressive presentations were common. Patients in this group more frequently had schizoid personality features.

**Conclusions:**

Endogenous affective disorders occurring with apathetic depression exhibit distinct dynamic features, which may serve as additional criteria for diagnosis and prognosis assessment.

**Disclosure of Interest:**

None Declared

